# Compounds with Antiviral, Anti-Inflammatory and Anticancer Activity Identified in Wine from Hungary’s Tokaj Region via High Resolution Mass Spectrometry and Bioinformatics Analyses

**DOI:** 10.3390/ijms21249547

**Published:** 2020-12-15

**Authors:** Gergő Kalló, Balázs Kunkli, Zoltán Győri, Zoltán Szilvássy, Éva Csősz, József Tőzsér

**Affiliations:** 1 Proteomics Core Facility, Department of Biochemistry and Molecular Biology, Faculty of Medicine, University of Debrecen, Egyetem tér 1, 4032 Debrecen, Hungary; kallo.gergo@med.unideb.hu (G.K.); cseva@med.unideb.hu (É.C.); 2 Biomarker Research Group, Department of Biochemistry and Molecular Biology, Faculty of Medicine, University of Debrecen, Egyetem tér 1, 4032 Debrecen, Hungary; 3 Laboratory of Retroviral Biochemistry, Department of Biochemistry and Molecular Biology, Faculty of Medicine, University of Debrecen, Egyetem tér 1, 4032 Debrecen, Hungary; kunkli.balazs@med.unideb.hu; 4 Doctoral School of Molecular Cell and Immune Biology, University of Debrecen, Egyetem tér 1, 4032 Debrecen, Hungary; 5 Institute of Food Science, Faculty of Agricultural and Food Sciences and Environmental Management, University of Debrecen, Böszörményi út 128, 4032 Debrecen, Hungary; gyori.zoltan@unideb.hu; 6 Department of Pharmacology and Pharmacotherapy, Faculty of Medicine, University of Debrecen, Egyetem tér 1, 4032 Debrecen, Hungary; szilvassy.zoltan@med.unideb.hu

**Keywords:** metabolomics, wine, LC-MS, functional food, high resolution mass spectrometry

## Abstract

(1) Background: Wine contains a variety of molecules with potential beneficial effects on human health. Our aim was to examine the wine components with high-resolution mass spectrometry including high-resolution tandem mass spectrometry in two wine types made from grapes with or without the fungus *Botrytis cinerea*, or “noble rot”. (2) For LC-MS/MS analysis, 12 wine samples (7 without and 5 with noble rotting) from 4 different wineries were used and wine components were identified and quantified. (3) Results: 288 molecules were identified in the wines and the amount of 169 molecules was statistically significantly different between the two wine types. A database search was carried out to find the molecules, which were examined in functional studies so far, with high emphasis on molecules with antiviral, anti-inflammatory and anticancer activities. (4) Conclusions: A comprehensive functional dataset related to identified wine components is also provided highlighting the importance of components with potential health benefits.

## 1. Introduction

In recent years, products of grapes have received great interest due to the discovery that several of their components have beneficial health effects on the human metabolism [1,2,3]. While grapes are carbohydrate-rich fruits, their glycemic index is quite low [4]. Furthermore, the polyphenol levels in grapes are relatively high and studies suggest the benefits of the polyphenol content of grapes and grape products in the prevention or treatment of cardiovascular diseases and diabetes [5]. Despite the great variety of fermented drinks made from fruits, grape wine is the most widespread fruit alcohol. Wine production from grape, i.e., vinification, is a multi-step process where each step plays an important role in the quality of the produced wine. The vinification process of red and white wines is different in the processing of the grape berries while the fermentation processes are similar [6,7]. In Hungary, wines produced in the Tokaj Hegyalja region, a declared UNESCO World Heritage site, are classified as Hungaricum [8]. “Furmint” is a traditional Hungarian grapevine variety, and one of the components of the world famous “aszú” wine [9]. “Tokaji aszú” is produced by a special process called noble rotting of the grapes. The procedure of noble rotting is an interaction between the enzymatic activity of *Botrytis cinerea* and the concentrating effect of the dehydration [8]. *Botrytis* produces various oxidase and hydrolase enzymes leading to the conversion of numerous grape components such as polyphenols, flavonoids and nitrogenous substances [10]. The noble rotten grape berries have high sugar content and unique chemical composition of acids, polyphenols and aroma compounds [10]. The berries are combined with high quality “furmint” wine and after another fermentation process, “aszú” wine will be produced that contains many of the compounds of the noble rotten grape berries produced and modified by the enzymes of *Botrytis cinerea* [10]. Moreover, “Tokaji aszú” is the first wine type utilizing noble rotting [11]. Besides “Tokaji aszú”, other type of wines are also utilizing the noble rotting process such as French Sauternes and German Trockenbeerenauslese wines [10].

The fact that wine was used as medicine in the ancient Mesopotamian and Chinese culture suggests the potential health benefits of wine components [12]. During the last decade, different studies have shown the health beneficial effects of wine polyphenols [13,14,15,16,17] suggesting that wine could be used as a functional food [12]. However, wine contains many other biologically important components that highlight the importance of the deep analysis of wine compounds.

The analysis of wine compounds includes different analytical techniques such as nuclear magnetic resonance (NMR) [18], infrared spectroscopy [8], gas chromatography-mass spectrometry (GC-MS) and liquid chromatography-mass spectrometry (LC-MS) [7]. The NMR technique is widely used for the analysis of the quality and the originality of different wines [18,19]. Although the NMR is very important for the quality control process of the winemaking procedure, the emerging mass spectrometry techniques can provide sensitive identification and quantification of hundreds of molecules in the wines. In spite of the recent improvements, the sensitivity is still a weak point of the NMR compared to mass spectrometry; the mass spectrometry-based metabolomics methods provide a more sensitive approach. Mass spectrometry enables the application of various ionization techniques and mass analyzers in order to increase the number of detected metabolites. With the application of mass spectrometry techniques, the differences between the wine types and wineries can be analyzed. In this study, our aim was to examine the wine components, to identify the components with health beneficial effects and to compare the small molecule content of different “furmint” and “aszú” wines originated from four wineries.

## 2. Results

In this study, we have analyzed 12 wine samples with data-dependent mass spectrometry acquisition using both positive and negative polarity modes in order to examine the different wine components. After data evaluation, 288 components were identified based on the acquired MS/MS spectra (Table S1). Based on the literature mining, 253 components were not reported in wines previously and these novel wine components were highlighted in Table S1. The identified compounds were subjected to comparative analysis using the normalized peak areas.

### 2.1. Biological Roles of the Identified Wine Compounds

The 288 identified compounds were subjected to analysis in the PubChem BioAssay database, and 137 molecules out of the 288 were found to have biological activity (Table S2). The collected PubChem identifiers were searched in the PUG-View database, as well. The acquired dataset was searched for compound description and information about biochemistry and human biochemistry, pharmacology, drug-medication and FDA safety hazard. In the PUG-View, 140 molecules out of the 288 hits were found (Table S3). In order to gain more data about the health beneficial effects of the identified molecules, the results of the search in the BioAssay database were further filtered to collect molecules with antiviral, anti-inflammatory and anticancer activity. Some highlights are given in Table 1, and detailed information is listed in Table S4.

### 2.2. Comparative Analysis of “aszú” and “furmint” Wines

**Figure 1:**
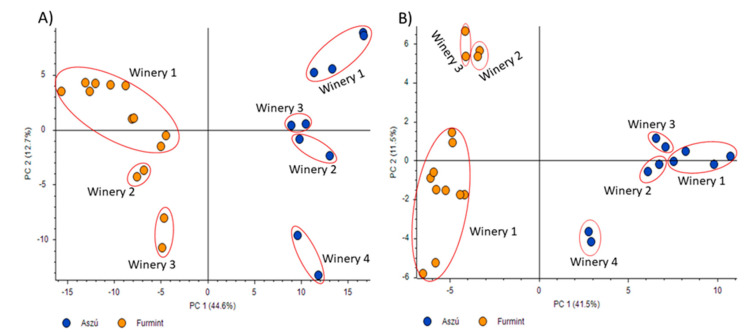
Principal component analysis of “aszú” and “furmint” wine samples analyzed in positive panel (**A**) and negative panel (**B**) polarity mode. The “x” axis shows PC1 while the “y” axis shows PC2. The orange dots represent the “furmint” samples while the blue dots represent the “aszú” samples. The wineries are also indicated with numbering.

Data acquired in positive and negative polarity modes were subjected to principal component analysis (Figure 1). Regarding the results, the differentiation between the “aszú” and “furmint” samples was successful using the data acquired both in positive (Figure 1A) and negative (Figure 1B) polarity modes. Besides the differentiation of the “aszú” and “furmint” wine types, we could also differentiate between the wineries using the data from positive polarity mode experiments (57.3%). In case of the data acquired in negative polarity mode, the differentiation of the wineries was not accurate enough (53%).

**Figure 2:**
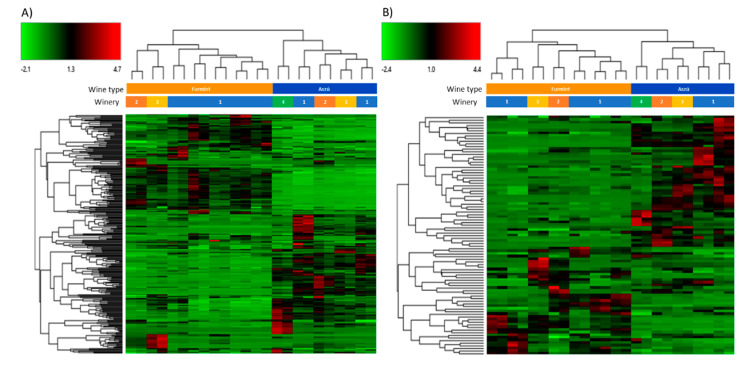
Hierarchical cluster analysis of the molecules identified in “aszú” and “furmint” wines registered in positive panel (**A**) and in negative panel (**B**) polarity mode. The green color indicates compounds with lower amount, while the red color shows compounds with higher amount in the comparison of “aszú” and “furmint” samples.

The acquired data were also subjected to hierarchical cluster analysis and heat maps were generated (Figure 2). The differentiation between the “aszú” and “furmint” wines was successful using the data registered in both positive (Figure 2A) and negative (Figure 2B) polarity mode. The “furmint” samples were clustered by the wineries in case of positive mode, while in negative polarity mode, the clustering was not as accurate as in positive mode. In case of the “aszú” samples, we observed that the clustering by wineries was successful in negative polarity mode, but not in positive mode. Based on the heat maps, several clusters of differentially expressed molecules between the two studied wine types could be identified.

**Figure 3:**
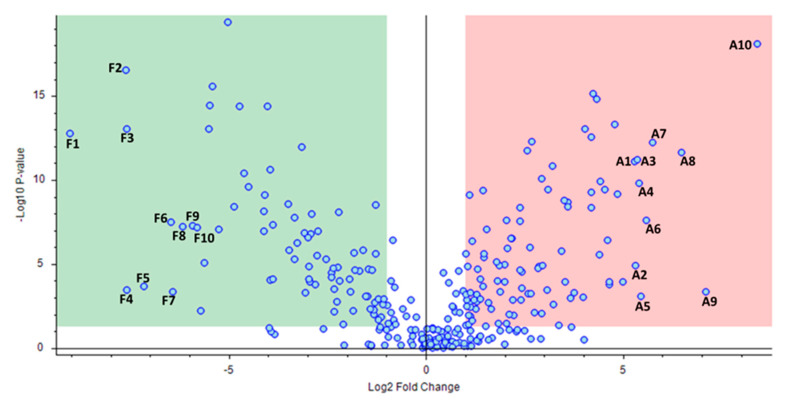
Comparative analysis of the identified compounds in “aszú” and “furmint” wines. The “x” axis represents the Log2 fold-change values, while the “y” axis indicates the –Log10 *p* values. The green box sows the compounds having statistically significantly higher level in “furmint” compared to “aszú”, while the red box indicates compounds with statistically significantly higher level in “aszú” compared to “furmint”. The top 10 components that showed the highest change in “furmint” and in “aszú”, repsepectively, are denoted with numbers (F1–F10 and A1–A10, respectively). F1: DL-isoleucyl-DL-isoleucyl-DL-histidine, F2: DL-alanyl-DL-isoleucyl-DL-isoleucyl-DL-threonine, F3: DL-leucyl-DL-leucyl-DL-leucine, F4: DL-valyl-DL-valyl-DL-valine, F5: L-phenylalanyl-L-leucine, F6: DL-tyrosyl-DL-prolyl-DL-isoleucine, F7: (2S)-2-[(2R)-7-(2-Methoxyethoxy)-5,8-dimethyl-1,2,3,4- tetrahydro-2-naphthalenyl]-1-(1-piperidinyl)-1-propanone, F8: DL-isoleucyl-DL-alpha-glutamyl-DL-lysine, F9: 1-(4-Methoxyphenyl)-N-[3-(4-morpholinyl)propyl]-5-oxo-3-pyrrolidinecarboxamide, F10: N-Isobutyrylglycylglycine, A1: Phenethylamine, A2: Perillartine, A3: DL-glutaminyl-DL-threonyl-DL-lysine, A4: N-Boc-Tyramine, A5: 4-(2,3-Dihydro-1,4-benzodioxin-6-yl)-4-oxobutanoic acid, A6: 1-Hydroxyhexane-1,2,6-tricarboxylate, A7: 2-Hydroxy-1-(6-hydroxy-2-isopropenyl-2,3-dihydro-1-benzofuran-5-yl)ethanone, A8: Rosin, A9: (2E)-3-(3,4-dimethoxyphenyl)prop-2-enoic acid, A10: N-[3-(4,11-Dimethyl-2-oxo-6,7,8,9-tetrahydro-2H-[1]benzofuro[3,2-g]chromen-3-yl)propanoyl]glycine.

After fold change analysis, 169 molecules with ±1 log2 fold change and with *p* < 0.05 between the “aszú” and “furmint” groups were identified (Figure 3, Table S5).

The top 10 molecules with the highest changes in “furmint” and top 10 molecules with the highest changes in “aszú” were further analyzed, and the biological roles of these molecules [20,21,22,23,24,25,26,27,28,29,30,31] are shown in Table 2.

## 3. Discussion

Appearing in ancient books, wine has been considered for a long time to be a preparation with health beneficial effects [12]. These effects are mainly attributed to the different phytochemicals present in wine, but there were also studies which demonstrated the beneficial effect of low doses of alcohol [32,33]. However, according to current studies, there are contradictory data regarding the beneficial or harmful effect of alcohol consumption [33]. Considering these data, we do not intend either to promote or discourage alcohol consumption; with this study, we aimed to carry out, with scientific rigorousness, a metabolomics examination of wine components followed by bioinformatics analysis.

The metabolomics analysis of “aszú” and “furmint” wine types identified altogether 288 different components. Although many common compounds were found in the two different wine types, we have identified 169 molecules characteristic either to “aszú” or “furmint”. The production of “furmint” and “aszú” wines are different, therefore, even though “aszú” is based on further processing and modification of “furmint” their composition is expected to be different, as well. In this pilot study, we have successfully differentiated the “aszú” wines involving berries having undergone noble rotting from the “furmint” wines based on the identified molecules and their relative quantities.

Phenolic compounds of the wine, including phenolic acids, flavonols, stilbenoids, dihydroflavonols, anthocyanins and flavanol monomers and polymers, influence the color and the taste of the wines. This large group of phenols can be separated into two broad groups, flavonoids and non-flavonoids [34,35]. Our analyses identified many phenolic compounds in the “aszú” and “furmint” wines such as 3-feruloylquinic acid, caffeic acid, caffeic acid 3-sulfate, chlorogenic acid, ethyl caffeate, dimethylcaffeic acid, ethyl gallate, fertaric acid, gentisic acid, rhododendrine and quercetin, and the compound annotation revealed that many identified phenolic compounds have antiviral, anticancer and anti-inflammatory activities. The statistical analysis has not shown statistically significant differences in the levels of caffeic acid, ethyl caffeate, ethyl gallate, fertaric acid, gentisic acid and quercetin between the “aszú” and “furmint” wines. The level of caffeic acid 3-sulfate and rhododendrine was significantly higher in “aszú” wines while the level of 3-feruloylquinic acid and chlorogenic acid was significantly higher in “furmint” wines. The reason behind the different level of several polyphenols in the different wines can be the additional fermentation process of the “aszú” wines and/or the infection of the grape berries with *Botrytis cinerea* [10].

Further studies are needed to gain more insights into the composition of wines, but our study has shown the power of LC-MS/MS-based metabolomics in wine examination. By applying the mass-spectrometry-based methods able to generate both qualitative and quantitative information, molecular fingerprints of different wines based on their compounds can possibly be created. By the bioinformatics analysis of the biological function of the wine components, we could generate comprehensive lists of the wine components highlighting their antiviral, anticancer and anti-inflammatory properties.

Viral outbreaks represent critical threat to the public health, particularly when vaccines or effective antiviral therapies are not available [36]. Viral infections can be responsible for significant global mortality and can be associated with several complex diseases such as Alzheimer’s disease, diabetes or cancer [37]. Herbal and fruit extract are well known sources of antiviral agents [37,38,39,40] providing new tools for the development of antiviral therapies. Phenolic compounds such as chlorogenic acid, ethyl caffeate, dimethylcaffeic acid, ethyl gallate, quercetin and taxifolin exert a wide range of antiviral activities against different viruses such as HIV-1 [41,42,43,44,45,46,47,48], hepatitis A, B and C virus [49,50,51,52,53,54], influenza virus [55,56,57,58,59,60,61], adenovirus [62,63], herpes simplex virus [62,63,64,65], enterovirus 71 [66,67], SARS-CoV [68], rhinovirus [69], Epstein–Barr virus [70] and coxsackievirus B4 [71] via different molecular mechanisms. Besides the phenolic compounds, the analysis of the mass spectrometry data from “aszú” and “furmint” wines revealed nine other components with proven antiviral activity, such as (E)-p-coumaric acid, 11-aminoundecanoic acid, coumarin, dehydrocostus lactone, flazin, indole-3-carbinol, melatonin, bestatin and umbelliferone. These compounds were already demonstrated by different research groups to have broad antiviral activities [72,73,74,75,76].

Viruses activate the immune system of the host that can further lead to inflammation [36]. The antiviral activity of the compounds described above mainly consists of the modulation of the immune response and initiating the inflammatory pathways. However, the proper balance between the pro-inflammatory and anti-inflammatory processes is required for the cells and organs to maintain their physiological functions. Thus, strictly regulated pro- and anti-inflammatory pathways are necessary for the homeostasis of the cells and tissues [77]. By database search and literature mining, we could identify 20 molecules in the “aszú” and “furmint” wine samples with anti-inflammatory activity (Table S4). From the 20 identified anti-inflammatory molecules, eight phenolic compounds, rhododendrin, caffeic acid, ethyl caffeate, chlorogenic acid, ethyl gallate, fertaric acid, quercetin and taxifolin were identified. Their anti-inflammatory effects involve the inhibition of toll-like receptor 4 and toll-like receptor 7 mediated signal transduction pathways [78,79], suppression of the NF-κB pathway [80,81,82,83,84,85,86], downregulation of COX-2 expression [87], a decrease in the level of inflammatory cytokines such as IL-1β, IL-6, IL-8, TNF-α and INF-γ [88,89,90], and reduced NO production [91]. In addition to phenolic components, tetrahydroharman-3-carboxylic acid, achalensolide, (E)-p-coumaric acid, zedoarondiol, asperlin, 9S, 13R-12-oxophytodienoic acid, dehydrocostus lactone, eicosapentaenoic acid, indole-3-carbinol, kynurenic acid, melatonin, and umbelliferone were also identified as molecules with anti-inflammatory activity. It is interesting to note that many of the compounds, such as umbelliferone, indole-3-carbinol, and melatonin, have both antiviral and anti-inflammatory activities at the same time, reflecting a complex mechanism of action [92,93,94,95].

Viral infections, inappropriate functioning of the immune system and other factors can lead the development of tumors [96]. The altered signaling pathways can lead to the dysregulation of the cell cycle and if the suppressor processes cannot work properly the cells lose their control on the replication machinery. Therefore, a strict anti-oncogenic regulation is required for the maintenance of the normal cell cycle [97]. From the 288 molecules identified in this study, 26 compounds have anticancer activity. Among these molecules, seven phenolic molecules were identified. Dimethylcaffeic acid can increase the level of polyamines in rats having an antitumor effect since the reduction in the level of polyamines is associated with cancer growth [98]. (E)-ethyl caffeate shows cytotoxicity against the different cancer cell lines [48], while ethyl gallate can promote apoptotic cell death [99,100]. Taxifolin can modulate the Nrf2 and Wnt/β-catenin cascade [101], can inhibit the proliferation of different cancer cell types [102], can initiate cell cycle arrest [103], and can activate apoptosis in prostate carcinoma cells [104]. Caffeic acid has anticancer activity against human cell lines originating from breast cancer [105,106], cervical cancer [106], metastatic cervical cancer [107], hepatocellular carcinoma [48], lung cancer, colon carcinoma [108], melanoma [109], oral squamous cell carcinoma [110], gastric cancer [111] and can suppress the UVB-induced skin carcinogenesis [112]. Besides antiviral, anti-inflammatory activities, quercetin also has anticancer activity against human cell lines originating from various cancer types [113,114,115,116,117,118,119,120,121,122,123,124], while chlorogenic acid is also a potent anticancer molecule with activity against human cell lines originating from different cancers [125,126,127,128,129]. We have also identified (E)-p-coumaric acid, 16-heptadecyne-1,2,4-triol, asperlin, 9S,13R-12-oxophytodienoic acid, coumarin, cyclo(phenylalanyl-prolyl), dehydrocostus lactone, DL-alanyl-DL-phenylalanine, dodecanedioic acid, eicosapentaenoic acid, indole-3-carbinol, sphinganine, L-histidinol, linamarin, melatonin, perlolyrine, phytosphingosine, pyridoxal and umbelliferone as molecules with proven anticancer activity.

The comprehensive collection of the biological functions of the identified wine components can provide a rich dataset to design in vitro and in vivo studies in order to test the beneficial effects of the different compounds. The data generated in this study can be used to design targeted examinations.

Validation of the wine components with health beneficial effects can provide high quality wines as functional food in the future. This can be especially important in case of many polyphenols such as quercetin, chlorogenic acid, caffeic acid, etc., which are insoluble in water but are soluble in alcoholic solutions. The alcohol content of wine helps the solvation of water insoluble polyphenols making wine a complex mixture of both water soluble and insoluble compounds such as phenolic compounds, acids, lipids, amino acids and other biologically active molecules [7]. The rich composition and the identified molecules with beneficial health effects highlight the potential of wine as a functional food [12].

## 4. Materials and Methods

### 4.1. Wine Samples

In this study, 12 wine samples from Tokaj Hegyalja region (Northeastern Hungary) were examined. Seven “furmint” and 5 “aszú” wines from 4 different wineries (winery 1: 2 “aszú” and 7 “furmint” samples, winery 2: 1 “aszú” and 1 “furmint” sample, winery 3: 1 “aszú” and 1 “furmint” sample, winery 4: 1 “aszú” sample) were subjected to analysis. The results of the routine chemical analysis according to Winsscan FTIR analysis (Foss Analytical A/S-HillerØd, Denmark) were listed in Table S6.

### 4.2. LC-MS Analysis

Prior to mass spectrometry analysis the components of the wines were separated using a Transcend II TLX-1 UPLC system (Thermo Scientific, Palo Alto, CA, USA) in LX mode. Chromatographic separations were performed on a TFS Hypersil gold reverse phase analytical column (50 × 2.1 mm, 1.9 μm particle size, 175 Å pore size, Thermo Scientific) using a 5 min water/acetonitrile gradient. The first step of the separation was a 25 s equilibration with 100% buffer A followed by the increase in solvent B to 30% during 5 s. Solvent B was further increased to 50% in 70 s and then increased to 95% in 60 s followed by a 60 s hold-on. In the last steps, the solvent condition was changed to 100% A in 30 s followed by the equilibration of the system with 100% solvent A. The flow rate was set to 0.8 mL/min. Solvent A was 0.1% formic acid in LC grade water (VWR Ltd., Radnor, PA, USA) and solvent B was 0.1% formic acid in LC grade acetonitrile (VWR Ltd.). The 100 µL samples were injected in duplicates.

Mass spectrometry analyses were performed on an Orbitrap Fusion tribrid mass spectrometer (Thermo Scientific) using data-dependent acquisition. Survey scans were taken in the Orbitrap mass analyzer with 120,000 mass resolution scanning a 100–1000 *m*/*z* range in profile mode. The AGC target was set to 4.0 × 10^5^. MS/MS spectra also were acquired in the Orbitrap mass analyzer by the fragmentation of the selected parent ions using HCD dissociation with 40% collision energy. However, MS/MS spectra were recorded in centroid mode at resolution and AGC target set to 50,000 and 5.0 × 10^4^, respectively. The cycle time of the analyses was 0.6 s. Spectra were acquired in both positive and negative polarity modes. The mass spectrometry data are available at the MassIVE database (ftp://MSV000085599@massive.ucsd.edu).

### 4.3. Data Analysis and Compound Identification

The acquired data were subjected to metabolite identification using the Compound Discoverer 3.1 software (Thermo Scientific). Both positive and negative polarity mode data were loaded for the analysis. For compound detection, the mass tolerance was set to 5 ppm, the intensity tolerance was 30%, the signal/noise ratio threshold was set to 3 and the minimum peak intensity was set to 100,000 cps. For the detection of compounds, {M+H]^+^, [M+Na]^+^, [M+K]^+^ and [M-H]^–^ ions were used. The grouping of the compounds was done with 5 ppm mass tolerance and 0.2 min retention time tolerance. The acquired MS2 spectra were searched against three different databases implemented into the Compound Discoverer for compound identification. The first database was the *m*/*z* cloud [130], the autoprocessed and reference libraries were used for the identification of small molecules. Besides the *m*/*z* cloud, the Chemspider database [131] was also used for identification. In the Chemspider, we used the Carotenoids database [132], FooDB [133], KEGG [134], LipidMAPS [135], Peptides [136], Phenol explorer [137], Plantcyc database [138] and the Yeast metabolome database [139]. The mass list library of the Compound Discoverer software was also used for compound identification. From the library, the Flavonoid structure database and the Endogenous metabolites database was selected for the search. The spectra of the identified molecules were analyzed manually and the hits were curated using the FISh scoring algorithm. For FISh scoring, the high mass accuracy tolerance was set to 2.5 Da while the low accuracy mass tolerance was 0.5 Da and the signal/noise threshold ratio was 3. The best hits were selected as compound annotations. Besides the identification, the comparative analysis of “aszú” and “furmint” wine types was also performed with the help of the software.

The peak areas were normalized by the software using constant mean normalization. For relative quantification the mean normalized peak areas were compared between the two studied groups. Principal component analysis implemented into the Compound Discoverer software was used considering the normalized peak areas. For heat map construction, normalized peak areas were used and the scaling was done before the clustering. The distance function was set to Euclidean and the linkage method was set to complete. For quantitative comparison of the “aszú” and “furmint” samples molecules with ±1 log2 fold change were accepted, and the significance threshold was set to *p* < 0.05.

### 4.4. Annotation of the Identified Compounds

In order to obtain information about the biological and pharmacological properties of the identified compounds, an in silico approach was implemented utilizing several in-house developed bash scripts to access ChemSpider, PubChem [140,141], and PubMed [142] databases via their programmatic web services. Based on the chemical names, first the ChemSpiderIDs, and SMILES identifiers were retrieved. SMILES strings were then included in PUG-REST web service [143] requests to access PubChem’s BioAssays [144] records and PubChem compound identifiers (CID) of each identified molecules. The retrieved CIDs were used to collect annotation data for each compound in the PubChem PUG-View interface [145]. CIDs were also used to collect literature metadata from PubMed by searching for PubMed IDs linked to the retrieved CIDs. The listed article titles, abstracts and BioAssays records were screened for specific keywords related to anti-inflammatory, antiviral and anticancer effects.

## Figures and Tables

**Table 1:** Antiviral, anticancer and anti-inflammatory roles of representative compounds found in wine. The full list of the compounds with detailed biological role and references are listed in Table S4.

Compound Name	PubChem ID	Role, Biological Activity
Achalensolide	21634938	NF-κB inhibitory activity in Jurkat T cells
DL-alanyl-DL-phenylalanine	2080	Cytotoxic effect against aggressive human metastatic breast adenocarcinoma MDA-MB-231 and MCF-7 cells
Asperlin	35319	Induction of apoptosis via ROS production in human cervical carcinoma HeLa cells
		Inhibition of iNOS, suppression of COX-2 expression, reduction in COX-derived PGE2
Bestatin	439299	Antiviral activity against coronaviruses
Caffeic acid	689043	Anticancer activity against human cell lines originating from breast cancer (MCF-7, MDA-MB-231) cervical cancer (HeLa), metastatic cervical cancer (SiHa), hepatocellular carcinoma (HepG2, Huh7), lung cancer (A-549), colon carcinoma (HT29-D4, HCT 15), melanoma (SK-Mel-28), oral squamous cell carcinoma (SCC-25), gastric cancer (SCM1), suppression of UVB-induced skin carcinogenesis
		Reduction in lipid peroxidation and of TNF-α, IL-6, IL-1β, IFN-γ, NF-κB/p65 and TGF-β levels
Dimethylcaffeic acid	717531	Antiviral activity against HIV-1
		Beneficial effect in prostate-, thymus- and stomach-, lung- and brain cancer
(E)-Ethyl caffeate	5317238	Antiviral activity against HBV, HIV-1
		Anticancer activity against cancer cell lines: human hepatocellular carcinoma BEL-7404 and HepG2, SK-OV-3 human ovarian cancer, human breast MCF-7 adenocarcinoma, human lung A549 adenocarcinoma and human gastric cancer BCG823
		Suppression of NF-κB activation and its downstream inflammatory mediators, iNOS, COX-2, and PGE2
Chlorogenic acid	1794427	Antiviral activity against hepatitis C, hepatitis B, Ebola virus, HIV-1, adenoviruses, H1N1/H3N2 influenza strains
		Anticancer activity against human cell lines originating from breast cancer (MCF-7, MDA-MB-231), cervical cancer (HeLa), hepatocellular carcinoma (HepG2), lung cancer (A-549), colon carcinoma (CT-26, Caco-2, HT29-D4, HCT 116), melanoma (SK-Mel-28), oral squamous cell carcinoma (HSC-2), salivary gland cancer (HSG), pancreatic cancer (PANC-1), leukemia (U937, HL-60, K562), prostate cancer (DU145)
		Anti-inflammatory activity, inhibition of NO and proinflammatory cytokine production
Coumarin	323	Coumarin derivatives exert anti-coagulant, anti-tumor, anti-viral, anti-inflammatory and antioxidant effects, as well as anti-microbial and enzyme inhibition properties
		Anticancer activity against human cell lines originating from gastric carcinoma, colon-carcinoma cell line (Caco-2), hepatoma-derived cell line (HepG2), lymphoblastic cell line (CCRF CEM), lung adenocarcinoma (A427, Calu-1, SK-MES-1, SK-LU-1), renal carcinoma (786-O, A-498), malignant prostatic cancer (DU145, LNCaP), beneficial effects in renal cell carcinoma and malignant melanoma
(E)-p-coumaric acid	637542	Antiviral activity against oseltamivir- and peramivir-sensitive and oseltamivir- and peramivir-resistant influenza viruses and hepatitis C
		Anticancer activity in mammalian, colon and hepatic cancer and neuroblastoma cell lines
		Inhibition of the STAT1 activation, decrease in TNF-α expression, ROS scavenger function
Cyclo(phenylalanyl-prolyl)	99895	Growth inhibition and apoptosis induction in HT-29 colon cancer cells
Dehydrocostus lactone	73174	Inhibition of Norovirus infection
		Anticancer activity against human cell lines originating from ovarian cancer (SK-OV-3), breast cancer (MCF-7, MDA-MB-231), cervical cancer (HeLa), hepatocellular carcinoma (HepG2), lung adenocarcinoma (A-549, NCI-H520, NCI-H460), prostate cancer (DU145), sarcoma (liposarcoma–SW-872, synovial sarcoma – SW-982, TE-671 – rhabdomyosarcoma), neuroblastoma (IMR-32, NB-39, SK-N-SH, LA-N-1)
		Reduced production of chemokines induced by TNF-α and IFN-γ
Dodecanedioic acid	12736	Cytotoxic effect on B16 melanoma cells
Eicosapentaenoic acid	446284	Growth inhibition effect on colon cancer cell lines (HT-29, Caco-2, DLD-1), antiproliferative effects on hepatoma (HepG2), leukemia (HL-60) cell lines, inhibition of macrophage-induced gastric cancer cell migration
		Attenuation of pro-inflammatory properties of VLDL via decrease in lipoprotein-lipase activity to hydrolyze VLDL
Ethyl gallate	13250	Anti-herpes simplex virus type 1 activity
		Anticancer activity against human cell lines originating from leukemia (HL-60), prostate cancer (PC-3), human (MCF-7) and mouse (S115) breast cancer, osteosarcoma (HOS-1), ovarian cancer (OVCAR-3), renal cancer (A-498), lung cancer (NCI-H460), colon cancer (KM20L2) and melanoma (SK-MEL-5)
		Inhibition of LPS induced cell adhesion molecules expression, attenuation of acute lung injury
Fertaric acid	22298372	Hepatoprotective effects
16-Heptadecyne-1,2,4-triol	3015189	Cytotoxic activity in human lung carcinoma (A-549), mammary adenocarcinoma (MCF-7), colon adenocarcinoma (HT-29), kidney carcinoma (A-498), pancreatic carcinoma (PaCa-2), prostate adenocarcinoma (PC-3) cell lines
L-Histidinol	165271	Pro-apoptotic activity in CCRF-CEM human leukemia cell line, inhibition of B16 melanoma cell proliferation
Indole-3-carbinol	3712	Promotion of apoptosis of Epstein–Barr virus (EBV)-positive but not of EBV-negative Burkitt’s lymphoma cell lines
		Anticancer activity against human cell lines originating from various cancer types such as prostate cancer (LnCaP, PC-3), breast cancer (MCF7, MDA-MB-468, MDA-MB-231, HBL100), colon cancer (HT-29, HCT-116), lung cancer (A-549), cervical cancer (CaSki, SiHa, C33-A), melanoma (SK-MEL-2, SK-MEL-5), ovarian cancer (SK-OV-3), oral squamous cell carcinoma (SCC2095, SCC9, SCC15), hepatocellular carcinoma (HepG2, Huh-7, SNU449), pancreatic cancer (BxPC-3, Mia Paca-2, PL-45, AsPC-1, PANC-1), leukemia (U937, HL-60, K562, BCP-ALL NALM-6), osteosarcoma (U2OS) etc.
		Reversal of liver fibrosis, reduction in hepatocyte degeneration, necrosis, promotion of hepatic stellate cell apoptosis, anti-inflammatory effects by inhibiting the productions of NO, TNF-alpha, and IL-10
Kynurenic acid	3845	Modulation of IL-23 and IL-17 expression in dendritic cells and Th17 cells
Linamarin	11128	Cytotoxic effects on MCF-7, HT-29 and HL60 cell lines
Melatonin	896	Indirect support against Ebola virus infection, Potential adjuvant treatment in COVID-19, and other viral infections
		Upregulation of Fas/Fas ligand in Ewing’s sarcoma cells, cell cycle arrest and apoptosis in hepatocarcinoma HepG2 cell line, induction of pro-apoptotic signaling pathway in human pancreatic carcinoma cells, anticancer activity in breast cancer (MCF-7) cells, inhibition of the proliferation and invasion of glioma cells, lung adenocarcinoma (A-549) cells, inhibition of estrogen receptor transactivation in breast cancer cells, negative mitogenic hormonal regulator of human prostate epithelial cells
		Decreases serum and tissue inflammatory cytokines levels, tissue lipid peroxidation and neutrophil infiltration
9S,13R-12-Oxophytodienoic acid	14037063	Induces growth arrest in MDA-MB-231 and T47D breast cancer cells followed by progressive reduction in cyclin D1 expression
		Suppression of NF-κB, inhibition of p38, and activation of SOCS-1 signaling
Perlolyrine	160179	Antiproliferative activity against human stomach cancer cell lines
Phytosphingosine	122121	Induction of apoptotic cell death via caspase 8 activation and Bax translocation in human cancer cells
Quercetin	5280343	Antiviral activity against HIV-1, hepatitis B and C viruses, adenoviruses, herpes simplex viruses, noroviruses, H1N1, H5N1 etc.
		Anticancer activity against human cell lines originating from various cancer types such as breast cancer (MCF7), colon cancer (HT-29, HCT-116, Caco-2, DLD-1), lung cancer (A-549), esophageal squamous cell carcinoma cell line (KYSE-510, OE33), cervical cancer (HeLa), oral squamous cell carcinoma (SCC9), hepatocellular carcinoma (HepG2), leukemia (U937, HL-60), osteosarcoma (MG-63), pancreatic cancer (PC3, EPP85-181P, EPP85-181RDB), melanoma (B16F10), glioma (U87, U139MG), inhibition of angiogenesis in tamoxifen-resistant breast cancer cells
		Inhibition of inflammatory cytokine production, Inhibition of histamine release, Reduction in neutrophil recruitment, Ameliorating endothelial insulin resistance through inhibition of reactive oxygen species-associated inflammation.
(-)-Rhododendrin	442538	Inhibition of toll-like receptor-7-mediated inflammation
Sphinganine	91486	Anticancer activity against human cell lines originating from leukemia (HL-60), prostate cancer (PC-3, LnCaP), breast cancer (MDA-MB-231), colon cancer (HT-29, HCT-116) and melanoma (939, 294, C8161, A2058), oral squamous cell carcinoma (SAS, Ca9-22, HSC-3)
Taxifolin	439533	Antiviral activity against HIV-1, coxsackieviruses B4, hepatitis A
		Anticancer effect due to Nrf2, inflammatory and Wnt/β-catenin cascade modulation, inhibition of breast cancer MDA-MB-231 and 4T1 cell proliferation, cell cycle arrest in human colorectal cancer HCT116 and HT29 cells, activation of apoptosis in prostate carcinoma DU145 cells
		Antioxidant and anti-inflammatory effects by inhibition of NO and PGE2 production, ICAM-1, COX-2 and PLA2 expression
Tetrahydroharman-3-carboxylic acid	73530	Inhibition of nitric oxide and prostaglandin E2 production
Umbelliferone	5281426	Antiviral activity against HIV-1
		Cytostatic activity in human malignant cell lines A549, ACHN, Caki-2, Dakiki, HS-Sultan, H727, HCT-15, HL-60, K562, LNCaP, PC-3, Du 145 COLO-232, MCF-7 and RP-1788; stimulation of apoptosis in HL-60 cells, Growth inhibition of human bladder carcinoma E-J cell line, lung adenocarcinoma A-427 cells, proliferation inhibition of gastric carcinoma, colon-carcinoma (Caco-2), a hepatoma-derived (HepG2), and a lymphoblastic cell line (CCRF CEM), chemoprotective effect in early-stage (Ln- Cap) and late-stage (PC3) prostate cancer cells
		Anti-inflammatory and antipyretic effects, reduction of IL-4, IL-5 and IL-13, suppression of Th1 cytokine production during influenza virus infection
Zedoarondiol	14632997	Inhibition of iNOS, COX-2 activity and of the production of NO, PGE2, TNF-alpha, IL-6, and IL-1beta

**Table 2:** Biological roles of the top 10 differentially expressed molecules in “aszú” and the top 10 differentially expressed molecules in “furmint” wines. The name, log2 fold change, adjusted *p*-value and the biological role is indicated in case of each compound. Negative fold change represents significantly higher level in “furmint”, while positive fold change shows significantly higher level in “aszú”.

Name	Log2 Fold Change	Adjusted *p*-Value	Biological Roles
DL-isoleucyl-DL-isoleucyl-DL-histidine	−9.04	2.43 × 10^−11^	No information
DL-alanyl-DL-isoleucyl-DL-isoleucyl-DL-threonine	−7.62	2.18 × 10^−14^	No information
DL-leucyl-DL-leucyl-DL-leucine	−7.60	1.43 × 10^−11^	No information
DL-valyl-DL-valyl-DL-valine	−7.58	1.35 × 10^−3^	No information
L-phenylalanyl-L-leucine	−7.16	8.81 × 10^−4^	Membrane metalloendopeptidase inhibitor in mouse model [20]
			Plant metabolite
DL-tyrosyl-DL-prolyl-DL-isoleucine	−6.46	5.77 × 10^−7^	No information
(2S)-2-[(2R)-7-(2-Methoxyethoxy)-5,8-dimethyl-1,2,3,4-tetrahydro-2-naphthalenyl]-1-(1-piperidinyl)-1-propanone	−6.42	1.76 × 10^−3^	No information
DL-isoleucyl-DL-alpha-glutamyl-DL-lysine	−6.18	9.00 × 10^−7^	No information
1-(4-Methoxyphenyl)-N-[3-(4-morpholinyl) propyl]-5-oxo-3-pyrrolidine carboxamide	−5.92	7.99 × 10^−7^	Phosphoethanolamine/phosphocholine phosphatase 1 inhibitor [21]
N-Isobutyrylglycylglycine	−5.79	1.04 × 10^−6^	No information
Phenethylamine	5.29	5.97 × 10^−10^	5-hydroxytryptamine receptor agonist in rat [22,23]
			CYP450 inhibitor [24]
			Trace amine associated receptor agonist [25]
Perillartine	5.32	7.90 × 10^−5^	No information
DL-glutaminyl-DL-threonyl-DL-lysine	5.37	4.92 × 10^−10^	No information
N-Boc-Tyramine	5.40	6.91 × 10^−9^	No information
4-(2,3-Dihydro-1,4-benzodioxin-6-yl)-4-oxobutanoic acid	5.45	2.92 × 10^−3^	Neuropeptide Y receptor agonist [26,27,28]
			Thyroid stimulating hormone receptor agonist [29]
1-Hydroxyhexane-1,2,6-tricarboxylate	5.58	4.60 × 10^−7^	No information
2-Hydroxy-1-(6-hydroxy-2-isopropenyl-2,3-dihydro-1-benzofuran-5-yl)ethanone	5.75	7.35 × 10^−11^	No information
Rosin	6.49	2.23 × 10^−10^	No information
(2E)-3-(3,4-dimethoxypheny)prop-2-enoic acid	7.11	1.77 × 10^−3^	Inhibitors of HSD17B4|hydroxysteroid (17-beta) dehydrogenase 4 [30]
			Antihemorrhagic activity in ddY mouse [31]
N-[3-(4,11-Dimethyl-2-oxo-6,7,8,9-tetrahydro-2H-[1]benzofuro[3,2-g]chromen-3-yl)propanoyl]glycine	8.42	1.57 × 10^−15^	No information

## Supplementary Materials

The following are available online at https://www.mdpi.com/1422-0067/21/24/9547/s1, Table S1: List of the compounds identified in “aszú” and “furmint” wines. The novel and previously annotated components identified in wine are highlighted in bold and italics, while the novel compounds without annotation are highlighted in bold, Table S2: Compound annotation based on the PubChem BioAssay database, Table S3: Compound annotation based on the PUG-View database, Table S4: Identified wine compounds with antiviral, anti-inflammatory and anticancer activity based on the BioAssay database and literature mining, Table S5: Compounds with significantly different levels in “aszú” and “furmint” wines, Table S6: Routine chemical analysis of the examined wines according to Winsscan FTIR analysis (Foss Analytical A/S-HillerØd, Denmark).

## Abbreviations

UNESCO	United Nations Educational, Scientific and Cultural Organization
NMR	Nuclear magnetic resonance
GC-MS	Gas chromatography-mass spectrometry
LC-MS	Liquid chromatography-mass spectrometry
MS/MS	Tandem mass spectrometry
FDA	Food and Drug Administration
LC-MS/MS	Liquid chromatography-tandem mass spectrometry
SARS-CoV	Severe acute respiratory syndrome coronavirus 2
COX-2	Cyclooxygenase-2
NF-κB	Nuclear factor-κB
IL-1β	Interleukin-1β
IL-6	Interleukin-6
IL-8	Interleunik-8
TNF-α	Tumor necrosis factor α
INF-γ	Interferon γ
NO	Nitrogen monoxide
Nrf2	Nuclear factor erythroid 2–related factor 2
CID	PubChem compound identifier
